# Evaluation of the Erosive and Cariogenic Potential of Over-the-Counter Pediatric Liquid Analgesics and Antipyretics

**DOI:** 10.3390/children8070611

**Published:** 2021-07-19

**Authors:** Eun-Ha Jung, Mi-Kyoung Jun

**Affiliations:** 1 Department of Dental Hygiene, Yonsei University, Wonju 26493, Korea; jeunha725@gmail.com; 2 Sae·e Dental Clinic, 109-8, Songwon-ro, Jangan-gu, Suwon 16294, Korea

**Keywords:** analgesics, antipyretics, dental caries, tooth erosion, pediatric dentistry

## Abstract

To evaluate the cariogenic and erosive potentials of over-the-counter pediatric oral liquid antipyretics and analgesics, we tested nine over-the-counter pediatric oral liquid medications classified as antipyretic or analgesic medicines available in Korea. For each substance, we measured the pH with a pH meter and the sugar content with a sugar content meter. We determined the titratable acidity (TA) levels based on the volumes of NaOH solution that had to be added to reach a pH of 7.0. We also evaluated the dental erosion potentials with an International Organization for Standardization method based on observing changes in the pH of a CaPO_4_ solution upon introducing a small volume of the solution to be tested. The oral liquid medications had pH values of 3.40–5.68. In the TA assessments, several oral liquid medications required greater volumes of NaOH solution to reach a pH of 7.0. The dental erosion potentials varied but correlated strongly with the NaOH volumes needed to reach a neutral pH (r = 0.84; *p* < 0.0001). Many oral liquid antipyretics and analgesics have features that can promote dental erosion. A correct understanding of pediatric antipyretics and analgesics is required in dentistry for children’s oral health.

## 1. Introduction

Dental erosion is defined as irreversible damage of the hard dental tissue due to chemical processes caused by acids that are not involved in bacterial action and may be associated with mechanical activities, such as abrasion and attrition [1]. Dental erosion could be due to intrinsic and/or extrinsic factors. Intrinsic factors, such as acid reflux and vomiting due to gastrointestinal diseases, and extrinsic factors are known to be related to the intake of foods with high acidic content and beverages, such as sports drinks, wines, and medicines, with acid as a constituent [2].

The initial stage of erosion on a tooth’s surface is the softening of the enamel surface, where the degree of damage varies depending on the type of acid and contact period. Without timely intervention, the erosion progresses and causes permanent damage to the tooth structure through the dissolution of enamel crystals [3]. Regarding its chemical significance, “enamel critical pH” is defined as a solution that is only saturated with respect to the minerals of the enamel, and the equilibrium state does not cause dissolution or mineral deposition of the tooth surface [4]. That is, the solution below the critical pH has a lower saturation [5] and there is a possibility of enamel demineralization.

Epidemiological studies on dental erosion in preschool children are being conducted worldwide, and its prevalence varies from 25 to 75% depending on the factors involved [4,5]. With the rapidly changing lifestyles, the consumption of beverages containing high concentrations of fermentable carbohydrates, such as carbonated drinks, energy drinks, and sports drinks, is increasing considerably, and thus dental erosion in children has emerged as a public health problem worldwide [6,7].

Besides these lifestyle changes, fever and cough are very common in pediatric and adolescent individuals. These ailments are managed with antipyretics in the form of syrups that are patient-friendly for this age group. The manufacturing process of syrups entails acid preparations to maintain drug dispersion and chemical stability to ensure physiological compatibility and improvement in flavor [8]. In addition to the acidic components, frequent intake, such as at least twice daily at bedtime and between meals; high viscosity; and other factors, such as additional effects, including saliva flow reduction, can contribute to increasing the risk of dental erosion [8]. In addition, additives, such as flavors and sweeteners, are used to minimize the unpleasant taste, such as the bitterness of the medicines [9]. Frequent intake of these syrups with a high sugar content, typically sucrose or fructose, or a combination of the two, may affect the pH of a dental biofilm. An acidic environment in the oral cavity below the critical pH (5.5) at which enamel demineralization occurs will lead to dental caries or erosion developing in the long term [10].

Institutions around the world recommend the reduction of sugar intake. The World Health Organization recommends sugars to be less than 10% of the total calorie intake [11]. The United States Food and Drug Administration indicates total sugar on the nutritional labeling attached to food and beverages and added sugars, such as sugar, syrup, and artificial honey. Indicating the quantity and the percentage of recommended daily intake on the packaging was mandated [12]. However, regulation of the type and content limit of the sugar used as an additive (sweetening agent) in the medicines is non-existent and is not considered when the medicine is prescribed.

Although published studies on the potential risks of dental caries due to medicine-related sugars or carbohydrates and their negative effects on the oral health of the children were evaluated [13], to date, research on the cariogenic and erosive effects of the over-the-counter (OTC) pediatric oral liquid medications are scarce. Therefore, the purpose of this study was to evaluate the cariogenic and erosive potentials of OTC pediatric oral liquid medications classified as antipyretics and analgesics.

## 2. Materials and Methods

### 2.1. Selection of Samples

A total of 9 OTC pediatric oral liquid medications classified as antipyretic and analgesic medicines sold on the Korean market were selected for each of the 3 types according to the 3 ingredients (Table 1). In addition, water (Jeju Samdasoo, Jeju Special Self-Governing Province Development Corporation, Jeju, Korea) was selected as a negative control and Yakult (Yakult, Korea Yakult Co., Ltd., Seoul, Korea) as a positive control for the comparison of erosion factors (Table 1). The raw material components of the medicine were recorded by referring to the items described through a search on the website of the Ministry of Food and Drug Safety. The purchased medicines were stored at room temperature according to the manufacturer’s recommended storage method and were used in the experiment immediately after opening.

### 2.2. Measurement of Acidity

The acidity of the experimental samples was measured using a calibrated pH meter (Orion 4 star; Thermo Orion, Beverly, CA, USA) while 100 mL of each of the test samples was stirred. The measurement was repeated three times and the results were calculated as an average value.

### 2.3. Measurement of the Buffering Capacity

The buffering capacity of the experimental samples was evaluated by examining the titratable acidity (TA) and measured by adding 1 M sodium hydroxide (NaOH) [14]. TA 5.5 and TA 7.0 were defined as the amount of 1 M NaOH required to reach a critical pH of 5.5, at which demineralization could occur on the tooth surface because of the experimental samples, and a neutral pH of 7.0, respectively. After measuring the baseline pH of the experimental samples, 1 M NaOH was added to check the change in pH. This addition of 1 M NaOH was continued until the pH value reached 10 and the required amount was recorded. The experimental samples were measured three times, as was done with the pH, and the result was calculated as an average value.

### 2.4. Evaluation of Dental Erosion Potential Using the International Organization for Standardization (ISO) Method

The dental erosion evaluation method proposed by the ISO was used to evaluate the erosion potential of the experimental samples. Calcium phosphate (CaPO_4_) solution (pH 5.05 ± 0.05) was used to mimic hydroxyapatite, which is the main component of tooth enamel [14]. The CaPO_4_ solution was made immediately before use and was used on the same day. After 25 mL of CaPO_4_ solution was stirred to measure the pH, 0.25 mL of the test sample was added and the pH was measured. The difference in pH before and after the addition of the test sample was defined as the amount of pH change (ΔpH). The calculated ΔpH is reported as the average of three measurements.

### 2.5. Measurement of Sugar Content 

The sugar content of the samples was measured with a sugar content meter (RHB-32, Lab & Tools, Gunpo, Korea). After placing 0.2 mL of the test agent on the prism, the sugar content was checked. The measured sugar content was recorded as brix%. In a sample with a brix value above the upper limit of the measurement range, the sample was diluted (1:10) and then the converted value was measured. The sugar content of the experimental group was measured 3 times and the average value was calculated.

### 2.6. Statistical Analysis

The potential for dental erosion between each group was evaluated using a one-way analysis of variance (ANOVA) followed by Scheffe post hoc analysis to identify statistically significant differences between the groups. The Spearman correlation coefficient was also calculated to evaluate associations between the pH and the dental erosion potential of fever reducers. All statistical analyses were performed using SPSS version 25 (IBM Corporation, Armonk, NY, USA), and the level of significance was set at *p* = 0.05.

## 3. Results

### 3.1. Acidity of Experimental Groups

The results of this experiment were calculated as the average value after a total of three evaluations for each of the groups. Yakult (positive control) had a pH of 3.59, and the pH of the mineral water (the negative control) was 7.79. The pH was lowest in Greenfen easy syrup (3.40) and highest in Maxibookids syrup (5.68) (Table 2). All of the experimental samples, except for the mineral water, exhibited acidic properties with a pH range of 3.40 to 5.38 (Table 2).

### 3.2. TA of Experimental Groups

The TA values of the liquid analgesics and antipyretics, according to the volume of 1 M NaOH added to evaluate the buffer capacity, are summarized in Table 2. The amount of NaOH (TA 5.5) required to reach a pH of 5.5 was the highest for Cokidsfen Syrup, followed by the Greenfen Syrup. In the TA evaluation for the Maxibookids Syrup that had the highest pH, the addition of NaOH did not reach TA 5.5. The highest amount of NaOH volume was needed to reach a TA 7.0 for Cokidsfen Syrup. Large volumes of NaOH were also required to achieve a pH of 7.0 in the following order: Greenfen Easy Syrup, Greenfen Syrup, and Children’s Brufen Syrup. These were higher than Yakult as the positive control group. Significant differences were found between the samples in both TA 5.5 and TA 7.0 (*p* < 0.05) (Table 2). The TA value for the mineral water, with pH > 7.0, could not be measured.

### 3.3. Evaluation of Erosive Potential According to the ISO Method

**Figure 1:**
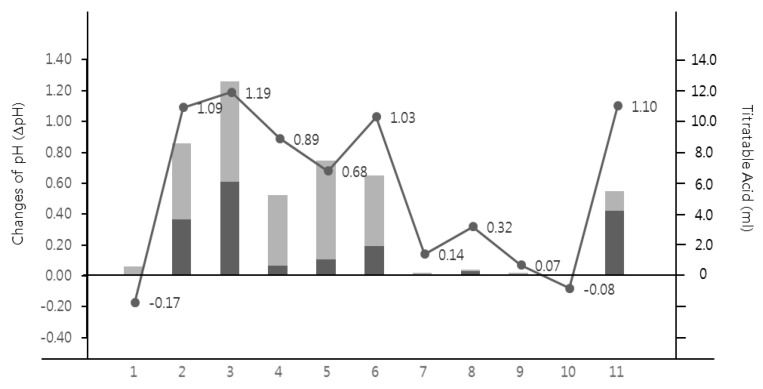
Test results for the erosive potential of fever reducers. The line indicates changes in pH of the CaPO_4_ solution after the addition of the experimental groups. The bar graph indicates the NaOH volume to obtain pH 5.5 and pH 7.0, respectively. The sample names were represented as follows; 1: Maxibookids Syrup, 2: Greenfen Easy Syrup, 3: Cokidsfen Syrup, 4: Champ Ibufen Syrup, 5: Greenfen Syrup, 6: Children’s Brufen Syrup, 7: Champ Syrup, 8: Coldaewon Kidsfen Syrup, 9: Children’s Tylenol Suspension, 10: Water, 11: Korea Yakult.

The differences between the initial pH and final pH of the CaPO_4_ solution after the addition of experimental samples were measured and reported as ΔpH values. As a result, the ΔpH values of the liquid analgesics and antipyretics were in the range of −0.17 to 1.19 (Table 2 and Figure 1). Greenfen Easy Syrup and Cokidsfen Syrup caused a higher change in the pH of the CaPO_4_ solution, and it was similar to Yakult (*p* < 0.05) (Table 2). In the case of Children’s Brufen Syrup, including these two samples showed △pH > 1 (Table 2). Regarding Maxibukids Syrup, the same change level was observed as with the mineral water (Table 2).

### 3.4. Sugar Content for Liquid Analgesics and Antipyretics

The sugar content range of 10–83 brix% was identified from the samples of liquid analgesics and antipyretics contained in the experimental groups, and significant differences between the groups were found (Table 2, *p* < 0.05). Between the groups, Maxibookids Syrup had the highest sugar content, followed by Children’s Brufen Syrup and Coldaewon Kidsfen Syrup. The sugar content of Greenfen Easy Syrup (10.03 brix%) was lower than that of the Yakult (18.30 brix%). In addition, all experimental groups, except for Greenfen Easy Syrup, exceeded the upper limit (50 brix%) of the measurement range (Table 2).

### 3.5. Relationship between pH and Erosive Potentials 

A high correlation was observed between the pH of the experimental groups and the erosive potential (TA 5.5, TA 7.0, and ∆pH). From this result, the TA and ∆pH from the CaPO_4_ solution were high according to the higher acidity of liquid analgesics and antipyretics. In particular, a very high negative correlation was observed between TA 5.5 and ∆pH. However, the sweetening agent concentration had a low correlation with pH (Table 3).

### 3.6. Relationship between Buffer Capacity and Dental Erosion

The association between the TA of the experimental groups and the dental erosion potential was evaluated and showed a very high positive correlation with both TA 5.5 and TA 7.0. A higher possibility of dental erosion was found when the liquid analgesics and antipyretics had great buffer capacities (0.974 and 0.840, respectively; *p* < 0.0001) (Figure 1).

## 4. Discussion

Many children frequently develop fevers because they are vulnerable due to the weaker immune system and thus have a higher susceptibility for infections [15]. With the recent COVID-19 pandemic, the uses of analgesics and antipyretics are increasing rapidly to resolve instances of high fever, which is one of the major symptoms of the infection [16]. In children with a fever, the average intake of analgesics and antipyretics was once every 8 h or 10 times a week [17]. For this reason, families prefer a child’s analgesics and antipyretics as a syrup or suspension [17]. Pediatric analgesics and antipyretics have a long history of use in the field of medicine. They are commonly prescribed, widely available, and easily accepted by children [18]. However, children’s analgesics and antipyretics contain citric acid for its antiseptic effects and large amounts of sugar for enhanced palatability (Table 1). After the intake of analgesics and antipyretics containing large amounts of citric acid and sugar, if the residues are present in the mouth for a long period, it can often cause dental caries and erosion in children [19]. Therefore, it is necessary to evaluate the ingredients of analgesics and antipyretics that are on the market for children and their potential for dental erosion so that an appropriate alternative can be presented.

All the evaluated analgesics and antipyretics had a pH range of 3.40–5.38, which is in the critical pH range (<5.5) for enamel demineralization (Table 2). The results were consistent with previous studies showing that most pediatric liquid medicines had acidic pHs [20,21]. This is expected because of the addition of citric acid to the analgesics and antipyretics (Table 1), which has a pH of 2.2 at 0.1 N and can cause dental erosion following long-term intake of such oral suspensions; citric acid increases enamel solubility owing to its ability to chelate calcium in the hydroxyapatite [22]. In addition, previous studies reported that enamel and dentin might dissolve at pH of 5.2–5.9 and 6.0–6.8, respectively [23,24]. This means that the teeth of children who frequently consume acidic liquid analgesics and antipyretics are at a high risk of dental erosion. To overcome this, efforts to recover the changed acid environment in the oral cavity above the critical pH level by minimizing the contact time of the medicine in the oral cavity after taking the analgesics and antipyretics are required.

TA is a more important indicator than pH because the acidic environment of the oral cavity can be recovered to neutral pH via the de-remineralization reaction [25]. First, 0.16–12.75 mL of NaOH was required for the experimental groups to reach a pH of 7.0. The TA value was higher than the positive control Yakult (5.57 mL) in the case of Cokidspen Syrup, Greenfen Easy Syrup, Greenfen Syrup, and Children’s Brufen Syrup (Table 2). The TA 5.5 measurement results showed that Cokidspen Syrup (6.14 mL), which required a greater amount of NaOH than the positive control Yakult (4.27 mL), had very high dental erosion potential. All experimental groups, except for the Cokidsfen Syrup, required approximately 0.00–3.67 mL of NaOH (Table 2). Considering that TA 5.5 is 5.76–16.02 mL of children’s drinks, TA 5.5 values in the experimental groups were considered acceptable [26]. Especially in the case of Maxibookid Syrup, Champ Ibufen Syrup, Champ Syrup, Coldaewon Kidsfen Syrup, and Children’s Tylenol Suspension showed a relatively low risk of dental erosion with a similar level of titratable acidity (for both TA 5.5 and TA 7.0). Nevertheless, care must be taken when taking a fever reducer because a fever reducer is made as a syrup or suspension solution type to dissolve and maintain the drug well. A previous study that evaluated the cariogenic potential index (CPI) according to the viscosity of the food confirmed that the CPI increases with higher viscosity [27]. Direct comparisons are difficult; it can be expected that the higher the viscosity, the more likely the acidic environment in the oral cavity will be maintained, which will also be associated with tooth erosion. In other words, it is important to be careful not to let anything in the mouth after taking a fever reducer, even if the level of titration is relatively low, given that syrup or a basal fluid form is more viscous than liquids such as water.

Despite the confirmation of the causation of dental erosion by pediatric antipyretic and analgesic syrups, the information provided to consumers is insufficient. This is thought to be due to the prioritization of antipyretic usage over the lack of consideration of the possible detrimental effects in the oral cavity. The International Organization for Standardization (ISO) recently proposed a method to compare the change in pH of CaPO_4_ solution by injecting the target products to assess the dental erosive potential of oral rinse. The CaPO_4_ solution was able to reproduce hydroxyapatite and was recently used to evaluate the dental erosion potential of various beverages [14]. In this study, the change in pH level of the CaPO_4_ solution was shown to have a very strong correlation with TA (r = 0.84–0.97, *p* < 0.0001) (Figure 1). This means that the method proposed by the ISO is appropriate for assessing the dental erosion of fever reducers, which can provide consumers with the necessary information through simple screening in the future. For the fever reducers included in this study, Cokidsfen Syrup, Greenfen Syrup, and Children’s Brufen Syrup showed significant changes at ΔpH 1 or higher, the same level as Yakult. The higher dental erosive potential was shown in a previous study, which indicated a similar or higher erosive risk with Sprite (ΔpH 1.0) and Coca-Cola (ΔpH 0.92), which are known as beverages with high erosive potential [14]. According to the ISO suggestions, the use of experimental solutions should be limited because the risk of dental erosion increases when the change in pH is >1.0 [14]. Therefore, efforts to minimize the oral damage caused by a low pH after taking fever reducers are necessary.

The relationship between pH, buffering capacity, and dental erosion possibility was analyzed to determine the effect of the pH of pediatric antipyretics on other erosive potential values. As a result, it was found that the higher the pH of the antipyretic agent and the buffering capacity, the higher the possibility of dental erosion (r = −0.89 and r = −0.75, respectively; *p* < 0.0001). However, it was confirmed that there was no relationship with sugar content. Considering that the sugar content of the antipyretic agent was up to 4.6 times higher than that of the Yakult as a positive control group, this study found that the sweetening agent in the fever reducer had less impact on dental erosion (Table 3). Nevertheless, the reason the amount of sugar in antipyretics should be considered is that regular use of liquid-type drugs containing sugar is often associated with the progression of dental caries in children. To evaluate the possibility of developing dental caries in children, the sugar content of the experimental medicine was measured and compared with the component table provided by the manufacturer. Eight types of the experimental medicines evaluated had fermenting carbohydrates and sucrose as the most common sweetening agents. Two types of sugars were mixed in seven experimental antipyretic agents, and some had higher amounts of sweeteners (Table 1). The sugar content of most of the experimental antipyretics (50.0 brix%) was higher than that of the Yakult (18.3 brix%), where the only exception was Greenfen Syrup (10.0 brix%). These results of the present study were slightly higher than the values obtained in a previous study (48.0 brix%) evaluating pediatric antipyretics [28] and were also higher than the carbonated beverages (12.2 brix%) [29]. It was already shown through the results of a previous study that the pH of the dental plaque decreases when a high-sugar-content medicine was taken [21]. In addition, excessive intake of sugar not only increases the risk of dental caries but also affects systemic health. Therefore, consideration is required regarding sugar intake [30]. There is no regulation of the ingredients and content of the sweetening agents added to the antipyretics, even though labeling of constituent nutrients of food products or beverages is mandatory in Korea. It is necessary to clearly present the sugar concentration for children’s dental and systemic health, thereby aiding in the selection of the medicines by well-informed consumers.

The limitation of this study was that the contents of calcium, phosphate, and fluorine compounds, which are known to be important factors in predicting the possibility of tooth erosion, were not quantified. In addition, the fact that only some of the antipyretic drugs currently available on the market in Korea were included in the study and limits the generalizability of the observations. However, this study is meaningful in that it considered the effects of antipyretics, which are frequently consumed by children, on dental health and suggests preventive measures. In future studies, we aim to expand the types of liquid medicines and evaluate them in consideration of other chemical aspects related to dental caries and erosion processes.

Considering this study’s results, the dental erosion and caries potential of OTC antipyretics were evident. Children’s dental health is a crucial component of their overall general health. Considering this, while taking a fever reducer, practicing precautionary measures, such as avoidance of intake before bedtime and emphasizing drug consumption at mealtime, minimizes tooth damage.

## 5. Conclusions

Most of the oral pediatric liquid analgesics and antipyretics included in the experiment showed that the pH value was lower than the threshold value and the buffering capacity was low. The potential for dental erosion and the sugar contents were high. These parameters were shown to increase the cariogenic and erosive potential of medicines. It is necessary to pay attention to the occurrence of dental caries and erosion caused by pediatric OTC antipyretic and analgesic usage. If long-term use is required, sugar-free or low-sugar content medicines should be suggested for children.

## Figures and Tables

**Table 1:** Experimental groups’ characteristics.

	Brand Name	Manufacturer	Clinical Composition	
			Sweetening Agent	Others
Experimental group	Maxibookids Syrup ^a^	Hanmi Pharm. Co., Ltd. (Seoul, Korea)	Sugar, high fructose corn syrup	Xanthan gum, citric acid hydrate, polysorbate 80, propyl p-hydroxybenzoate, betadex, D-sorbitol solution, methyl p-hydroxybenzoate, sodium citrate hydrate, sodium benzoate, magnesium aluminosilicate, agar
	Greenfen Easy Syrup ^a^	Mcnulty Pharmaceutical (Chungcheongnam-do, Korea)	Sucralose, high fructose corn syrup, acesulfame potassium	Xanthan gum, citric acid hydrate, polysorbate 80, propyl p-hydroxybenzoate, concentrated glycerin, methyl p-hydroxybenzoate, sodium citrate hydrate, mixed fruit flavor, sodium benzoate, agar
	Cokidsfen Syrup ^a^	Kolon Pharmaceuticals, Inc. (Seoul, Korea)	Sugar, sucralose	Xanthan gum, citric acid hydrate, sodium benzoate, cellulose microcrystalline and carboxymethylcellulose, propyl p-hydroxybenzoate, concentrated glycerin, sodium citrate hydrate, D-sorbitol solution, polysorbate 80, methyl p-hydroxybenzoate, kaolin light
	Champ Ibufen Syrup ^b^	Dong A Pharmaceutical Co., Ltd. (Seoul, Korea)	Sugar, sucralose	D-sorbitol solution, concentrated glycerin, cellulose microcrystalline and carboxymethylcellulose, citric acid, xanthan gum, polysorbate 80, propylene glycol
	Greenfen Syrup ^b^	GC Pharma Korea (Gyeonggi-do, Korea)	Xylitol, steviol glycoside	Orange essence, sodium chloride, citric acid hydrate, propyl p-hydroxybenzoate, carboxymethylcellulose, agar, glycerin, sodium citrate hydrate, magnesium aluminum silicate, polysorbate 80, methyl p-hydroxybenzoate
	Children’s Brufen Syrup ^b^	Samil Pharmaceutical Co., Ltd. (Seoul, Korea)	Sugar	Citric acid hydrate, propyl p-hydroxybenzoate, concentrated glycerin, polysorbate 80, methyl p-hydroxybenzoate, sodium benzoate, kaolin, sodium citrate hydrate, agar, D-sorbitol solution
	Champ Syrup ^c^	Dong-A Pharmaceutical Co., Ltd. (Seoul, Korea)	Sugar, sucralose	D-sorbitol solution, citric acid, propylene glycol, xanthan gum, cellulose microcrystalline and carboxymethylcellulose, concentrated glycerin
	Coldaewon Kidsfen Syrup ^c^	Daewon Pharmaceutical Co., Ltd. (Seoul, Korea)	Sugar, sucralose	Cellulose microcrystalline and carboxymethylcellulose, xanthan gum, povidone, citric acid, propylene glycol, concentrated glycerin, citrus flavor, D-sorbitol solution
	Children’s Tylenol Suspension ^c^	Janssen Korea Co., Ltd. (Seoul, Korea)	Sucrose, sucralose, sorbitol solution 70%	Propylene glycol, propyl p-hydroxybenzoate, bitter mask, anhydrous citric acid, glycerin, cellulose microcrystalline and carboxymethylcellulose, butyl paraoxybenzoate, xanthan gum
Negative control group	JeJu Samdasu	Kwang Dong Pharmaceutical Co., Ltd. (Seoul, Korea)	-	Bedrock groundwater
Positive control group	Yakult	Korea Yakult Co., Ltd. (Kyeonggi-do, Korea)	Sucralose	Sodium, carbohydrate, saccharide, sugar alcohol, protein, calcium

^a^: Dexibuprofen, ^b^: Ibuprofen, ^c^: Acetaminophen.

**Table 2:** Potential for dental erosion from liquid analgesics and antipyretics.

Brand Name	pH	Titratable Acid (mL)		∆pH	Brix%
		TA5.5	TA7.0		
Maxibookids Syrup	5.68 ± 0.02 ^a^	0.00 ± 0.00 ^a^	0.59 ± 0.02 ^a^	−0.17 ± 0.04 ^a^	83.30 ± 2.89 ^a^
Greenfen Easy Syrup	3.40 ± 0.01 ^b^	3.67 ± 0.29 ^b^	8.69 ± 0.33 ^b^	1.09 ± 0.01 ^b,c^	10.03 ± 0.06 ^b^
Cokidsfen Syrup	3.78 ± 0.01 ^c^	6.14 ± 0.27 ^c^	12.75 ± 0.29 ^c^	1.19 ± 0.02 ^c^	50.67 ± 0.58 ^c^
Champ Ibufen Syrup	3.80 ± 0.02 ^c^	0.65 ± 0.01 ^d,e^	5.29 ± 0.20 ^d^	0.89 ± 0.04 ^d^	63.00 ± 0.00 ^d^
Greenfen Syrup	4.52 ± 0.04 ^d^	1.08 ± 0.04 ^e^	7.57 ± 0.39 ^e^	0.68 ± 0.03 ^e^	51.00 ± 1.00 ^c^
Children’s Brufen Syrup	3.85 ± 0.01 ^c^	1.96 ± 0.06 ^f^	6.60 ± 0.26 ^f^	1.03 ± 0.01 ^b^	80.00 ± 0.00 ^a,e^
Champ Syrup	4.79 ± 0.02 ^e^	0.09 ± 0.01 ^a^	0.19 ± 0.01 ^a^	0.14 ± 0.03 ^f^	70.67 ± 0.58 ^f^
Coldaewon Kidsfen Syrup	4.41 ± 0.06 ^d^	0.28 ± 0.03 ^a,d^	0.38 ± 0.03 ^a^	0.32 ± 0.06 ^g^	79.00 ± 1.00 ^e^
Children’s Tylenol Suspension	5.25 ± 0.00 ^f^	0.04 ± 0.01 ^a^	0.16 ± 0.00 ^a^	0.07 ± 0.01 ^f^	62.00 ± 1.00 ^d^
Water	7.79 ± 0.05 ^g^	0.00 ± 0.00 ^a^	0.00 ± 0.00 ^a^	−0.08 ± 0.03 ^a^	0.00 ± 0.00 ^g^
Korea Yakult	3.59 ± 0.02 ^h^	4.27 ± 0.02 ^g^	5.57 ± 0.02 ^d^	1.10 ± 0.01 ^b,c^	18.30 ± 0.00 ^h^

Values are presented as mean ± standard deviation. TA: titratable acidity. ^a–h^ Different letters within the same column indicate significant differences between groups according to Scheffe post hoc analysis at alpha = 0.05.

**Table 3:** Correlation (r values) between the pH and dental erosion values from liquid analgesics and antipyretics.

pH	Titratable Acid		∆pH	Brix
	TA5.5	TA7.0		
r	−0.891 *	−0.752 *	−0.918 *	0.251
*p*	<0.0001	<0.0001	<0.0001	0.158

* *p* < 0.01.

## Data Availability

Not applicable.

## References

[1] Jarvinen V., Rytomaa I., Heinonen O. (1991). Risk factors in dental erosion. J. Dent. Res..

[2] Scheutzel P. (1996). Etiology of dental erosion–intrinsic factors. Eur. J. Oral Sci..

[3] Lussi A., Schlüter N., Rakhmatullina E., Ganss C. (2011). Dental erosion–an overview with emphasis on chemical and histopathological aspects. Caries Res..

[4] Dawes C. (2003). What is the critical pH and why does a tooth dissolve in acid?. J. Can. Dent. Assoc..

[5] Alves L.S., Brusius C.D., Damé-Teixeira N., Maltz M., Susin C. (2015). Dental erosion among 12-year-old schoolchildren: A population-based cross-sectional study in South Brazil. Int. Dent. J..

[6] Zhang S., Chau A.M., Lo E.C., Chu C.-H. (2014). Dental caries and erosion status of 12-year-old Hong Kong children. BMC Public Health.

[7] Mantonanaki M., Koletsi-Kounari H., Mamai-Homata E., Papaioannou W. (2013). Dental erosion prevalence and associated risk indicators among preschool children in Athens, Greece. Clin. Oral Investig..

[8] Scatena C., Galafassi D., Gomes-Silva J.M., Borsatto M.C., Serra M.C. (2014). In vitro erosive effect of pediatric medicines on deciduous tooth enamel. Braz. Dent. J..

[9] Mennella J.A., Spector A.C., Reed D.R., Coldwell S.E. (2013). The bad taste of medicines: Overview of basic research on bitter taste. Clin. Ther..

[10] Durward C., Thou T. (1997). Dental caries and sugar-containing liquid medicines for children in New Zealand. N. Z. Dent. J..

[11] Nishida C., Uauy R., Kumanyika S., Shetty P. (2004). The joint WHO/FAO expert consultation on diet, nutrition and the prevention of chronic diseases: Process, product and policy implications. Public Health Nutr..

[12] Hu F.B., Ave H. (2016). The Revised Nutrition Facts Label A Step Forward and More Room for Improvement. JAMA.

[13] Donaldson M., Goodchild J.H., Epstein J.B. (2015). Sugar content, cariogenicity, and dental concerns with commonly used medications. J. Am. Dent. Assoc..

[14] Kim S.K., Park S.W., Kang S.M., Kwon H.K., Kim B.I. (2015). Assessment of the erosive potential of carbonated waters. J. Korean Acad. Oral Health.

[15] Kim S.Y. (2003). Evidence-based upper respiratory infection prescription. J. Korean Med Assoc..

[16] Fialkowski A., Gernez Y., Arya P., Weinacht K.G., Kinane T.B., Yonker L.M. (2020). Insight into the pediatric and adult dichotomy of COVID-19: Age-related differences in the immune response to SARS-CoV-2 infection. Pediatric Pulmonol..

[17] Sullivan J.E., Farrar H.C. (2011). Fever and antipyretic use in children. Pediatrics.

[18] Babu K.G., Doddamani G.M., Naik L.K., Jagadeesh K. (2014). Pediatric liquid medicaments–are they cariogenic? An in vitro study. J. Int. Soc. Prev. Community Dent..

[19] Cavalcanti A.L., Fernandes L.V., Barbosa A.S., Vieira F.F. (2008). pH, Titratable Acidity and Total Soluble Solid Content of Pediatric Antitussive Medicines. Acta Stomatol. Croat..

[20] Tupalli A.R., Satish B., Shetty B.R., Battu S., Kumar J.P., Nagaraju B. (2014). Evaluation of the erosive potential of various pediatric liquid medicaments: An in-vitro study. J. Int. Oral Health.

[21] Saeed S., Bshara N., Trak J., Mahmoud G. (2015). Effect of dietary combinations on plaque pH recovery after the intake of pediatric liquid analgesics. Eur. J. Dent..

[22] Reddy A., Norris D.F., Momeni S.S., Waldo B., Ruby J.D. (2016). The pH of beverages available to the American consumer. J. Am. Dent. Assoc..

[23] Meurman J., Ten Gate J. (1996). Pathogenesis and modifying factors of dental erosion. Eur. J. Oral Sci..

[24] Zimmer S., Kirchner G., Bizhang M., Benedix M. (2015). Influence of various acidic beverages on tooth erosion. Evaluation by a new method. PLoS ONE.

[25] Benjakul P., Chuenarrom C. (2011). Association of dental enamel loss with the pH and titratable acidity of beverages. J. Dent. Sci..

[26] Hunter L., Patel S., Rees J. (2009). The in vitro erosive potential of a range of baby drinks. Int. J. Paediatr. Dent..

[27] Lee K.-S., Kim N.-J., Lee E.-H., Cho J.-W. (2014). Cariogenic potential index of fruits according to their viscosity and sugar content. Int. J. Clin. Prev. Dent..

[28] Xavier A.F.C., Moura E.F., Azevedo W.F., Vieira F.F., Abreu M.H., Cavalcanti A.L. (2013). Erosive and cariogenicity potential of pediatric drugs: Study of physicochemical parameters. BMC Oral Health.

[29] Jun M.-K., Lee D.-H., Lee S.-M. (2016). Assessment of nutrient and sugar content and ph of some commercial beverages. J. Dent. Hyg. Sci..

[30] Paglia L. (2019). The sweet danger of added sugars. Eur. J. Paediatr. Dent.

